# Lipoprotein SPD_1609 of *Streptococcus pneumoniae* Promotes Adherence and Invasion to Epithelial Cells Contributing to Bacterial Virulence

**DOI:** 10.3389/fmicb.2019.01769

**Published:** 2019-07-30

**Authors:** Xiao-Yan Yang, Nan Li, Jing-Yu Xu, Xuesong Sun, Qing-Yu He

**Affiliations:** ^1^Zhuhai Key Laboratory of Basic and Applied Research in Chinese Medicine, Department of Bioengineering, Zhuhai Campus of Zunyi Medical University, Zhuhai, China; ^2^Key Laboratory of Functional Protein Research of Guangdong Higher Education Institutes, Institute of Life and Health Engineering, College of Life Science and Technology, Jinan University, Guangzhou, China

**Keywords:** *Streptococcus pneumoniae*, SPD_1609, iron uptake, adherence, invasion, bacteremia infection

## Abstract

In most bacteria, iron plays a vital role in pathogenesis processes. To support survival and infection, *Streptococcus pneumoniae* has evolved three ABC transporters, PiaABC, PiuABC, and PitABC. Moreover, indirect evidence supports that operon 804 may be a novel ABC transporter in *S. pneumoniae*. We have recently described the identification of lipoprotein SPD_1609 in operon 804; however, whether the SPD_1609 protein affects the virulence of *S. pneumoniae* has not yet been studied. In the present work, alignment analysis showed that lipoprotein SPD_1609 is conserved in a variety of gram-positive bacteria, and deletion of the *spd_1609* gene led to a reduction in adherence and invasion of *S. pneumoniae* to host cells. RT-qPCR assays indicated that deletion of the *spd_1609* gene resulted in decreased expression of genes involved in colonization. Furthermore, decreased virulence in a mouse bacteremia infection model caused by the loss of the lipoprotein encoded by *spd_1609* was also demonstrated. Overall, these data provide insights into the functional role of lipoprotein SPD_1609 in pneumococcal pathogenesis, suggesting its possibility to be developed as a novel *S. pneumoniae* vaccine candidate.

## Introduction

*Streptococcus pneumoniae* (*S. pneumoniae*) is a gram-positive human pathogen and a frequent inhabitant of the upper airways in healthy hosts. *S. pneumoniae* can invade the lower airways when the immune system is weakened, causing a variety of serious diseases including pneumonia, bacterial meningitis, sinusitis, otitis media and bacteremia that are threats to public health (Van Der Poll and Opal, 2009). To survive and establish infection in the host, *S. pneumoniae* must acquire essential nutrients, including transition metals iron, manganese, zinc, and copper (Honsa et al., 2013).

Iron concentrations ranging from 10^–7^ to 10^–5^ M are required by most bacteria for various biological processes, including glycolysis, oxygen transport, gene regulation, and DNA biosynthesis (Andrews et al., 2003; Schaible and Kaufmann, 2004; Cassat and Skaar, 2013). However, the concentration of free iron in the host environment is extremely low (∼ 10^–17^ M) (Andrews et al., 2003). As a consequence, many bacteria have developed multiple specialized iron uptake mechanisms to acquire iron from the host, such as the production of siderophores, heme acquisition systems, ferric or ferrous iron transporters, transferrin or lactoferrin receptors, and utilization of manganese instead of iron in metalloenzymes (in *Borrelia burgdorferi*) (Cassat and Skaar, 2013).

Iron plays a crucial role in the pathogenesis processes of *S. pneumoniae* (Honsa et al., 2013; Turner et al., 2017). *S. pneumoniae* can utilize heme, hemoglobin, ferrichrome, ferric and ferrous iron as iron sources, but not lactoferrin or transferrin (Romero-Espejel et al., 2013; Turner et al., 2017). To support survival and infection, *S. pneumoniae* has evolved three ABC transporters, PiaABC, PiuABC, and PitABC, with lipoproteins PiaA, PiuA, and PitA as substrate-binding proteins to uptake iron (Brown et al., 2001a, 2002; Yang et al., 2016). Moreover, indirect evidence supports that operon 804 may encode a novel ABC transporter in *S. pneumoniae* (Yang et al., 2016).

There are reports showing that loss of bacterial iron transporter systems often corresponds with affected virulence in murine models (Brown et al., 2001a; Torres et al., 2006; Pishchany et al., 2014). In *Staphylococcus aureus*, a mutant lacking IsdB (cell wall-anchored protein of iron-regulated surface determinant system Isd) shows a reduced virulence in a murine model of abscess formation (Torres et al., 2006; Pishchany et al., 2014). In *S. pneumoniae*, Brown et al., 2001a have reported that the *piaA*- mutant exhibits reduced virulence in both mouse systemic and pulmonary infection models, and the *piuB*- and *pitA*- mutants exhibit reduced virulence only in a systemic infection model (Brown et al., 2001a, 2002).

Bacterial lipoproteins are a major category of membrane proteins with various functions. These proteins often have important effects on pathogen/host interactions during the development of bacterial infection, and thus some of them have been shown to be potential vaccines (Brown et al., 2001b; Hutchings et al., 2009; Kovacs-Simon et al., 2011; Kohler et al., 2016; Nguyen and Gotz, 2016; Yang et al., 2016). In gram-positive bacteria, most of these lipoproteins are substrate-binding proteins of ABC transporters involved in the transport of a set of substrates such as metal ions, amino acids, peptides, sugars, lipids and vitamins that are necessary for virulence (Brown et al., 2001b; Kohler et al., 2016; Yang et al., 2016). We recently reported that lipoprotein SPD_1609 in operon 804 was involved in iron uptake; however, whether the SPD_1609 protein affects the virulence of *S. pneumoniae* has not yet been studied (Yang et al., 2016). Therefore, the aim of this work was to investigate the effects of lipoprotein SPD_1609 on pneumococcal virulence *in vitro* and *in vivo*.

## Materials and Methods

### Ethics Statement

Balb/c mice were supplied by the Laboratory Animal Unit of Sun Yat-sen University. All animal experiments were approved by the Committee on the Use of Live Animals in Teaching and Research of Zunyi University and conformed to institutional and governmental guidelines and regulations.

### Bacterial Strains and Growth Conditions

All *S. pneumoniae* strains used in this study were derivatives of the parental *S. pneumoniae* D39 strain. These *S. pneumoniae* strains were cultured in Todd-Hewitt broth (Oxoid, United Kingdom) containing 0.5% yeast extract (THY) or grown on Columbia agar (Difco, United States) containing 5% sheep blood (Ruite, China) at 37^∘^C in a 5% CO_2_ incubator. *Escherichia coli* DH5α was grown in LB medium (1% tryptone, 0.5% yeast extract, 1% NaCl) at 37^∘^C with shaking at 200 rpm. When required, growth media for *S. pneumoniae* were supplemented with tetracycline (Tet, 3.5 μg/mL) and chloramphenicol (Cm, 4 μg/mL), and media for *E. coli* were supplemented with ampicillin (Amp, 100 μg/mL) and chloramphenicol (Cm, 20 μg/mL). The iron-restricted medium was prepared by adding 5% Chelex-100 (Bio-Rad, United States) to THY for 8 h under continuous stirring, then filtering sterilization to remove the Chelex-100 and supplementing with 100 μM CaCl_2_ and 2 mM MgCl_2_. When necessary, 20 μM FeCl_3_, hemin or ferrichrome (Fch) was added to the iron-restricted medium.

### Multiple Amino Acid Sequence Alignment

Comparative analysis between the amino acid sequence of *S. pneumoniae* SPD_1609 protein without the signal peptide (35−355 AA) with multiple protein sequences of (iron) ABC transporter substrate-binding proteins from *Streptococcus pseudopneumoniae*, *Streptococcus mitis*, *Streptococcus infantis*, *Streptococcus suis*, *Clostridium cadaveris*, *Abiotrophia defectiva*, *Granulicatella adiacens*, *Hungatella hathewayi*, *Roseburia intestinalis*, *Bacillus cereus*, *Paenibacillus* sp. *Y412MC10*, *Bacillus* sp. *FJAT-14515*, *Trueperella pyogenes*, and *Lactobacillus heilongjiangensis* and the PitA protein of *S. pneumoniae* TIGR4 were performed. The sequence similarity searches of SPD_1609 were initiated by a Position-Specific Iterated BLAST (PSI-BLAST) search and analyzed by Clustal X2.1. To reflect exactly the similarity of these proteins, the alignment was performed only with the mature part of the lipoprotein, without the signal peptide, and the signal peptide was predicted using Signal P 4.1 soft.

### Construction of the *1609*- Mutant Strain and the *1609* Complement Strain

A long flanking homology-polymerase chain reaction (LFH-PCR) process was used to generate the *spd-1609*- mutant strain (*1609*- mutant) (Wach, 1996). Briefly, the 500 bp region upstream of *spd-1609* was amplified using primers *spd-1609*–P1 and *spd-1609*–P3, while the 500 bp region downstream of *spd-1609* was amplified using primers *spd-1609*–P2 and *spd-1609*–P4. The tetracycline gene was amplified using primers *tet-F* and *tet-R*. The three PCR fragments generated were joined together by overlap extension PCR using primers *spd-1609*–P1 and *spd-1609*–P2 to form an approximately 2.5-kb final linear DNA construct containing the deletion fragment. Then, linear DNA construction was used for homologous recombination and transformed into *S. pneumoniae* D39. Transformants were selected with agar plates containing 3.5 μg/mL tetracycline, and mutants were confirmed by PCR and Western blotting. All mutations were stable after six sequential passages in 0.5% THY without antibiotic selection. The primers used for the construction of the mutants are listed in Supplementary Table S1.

To confirm the link between the *1609*- mutant strain and the phenotype exhibited by the *S. pneumoniae 1609*- mutant, a complement strain (*1609* complement) that contains a recombinant shuttle plasmid pIB169 (Biswas et al., 2008) with the coding sequence of the full-length *spd-1609* gene in the isogenic *1609*- mutant background was used. The full *spd-1609* gene, including a C-terminal 6 × His-tag, was amplified with primers pIB169-*1609*-F and pIB169-*1609*-R by PCR from the D39 genomic DNA, digested with *Sac* II and *Kpn* I and then ligated into the digested vector to generate the complementation plasmid, pIB169-*1609*. The recombinant plasmid was screened by using 20 μg/mL Cm in *E. coli* and 4 μg/mL Cm in *S. pneumoniae* and confirmed by PCR, DNA sequencing and Western blotting.

### Determination of Growth Curves

*Streptococcus pneumoniae* D39 wild type, *1609*- mutant and *1609* complement strains were inoculated into normal THY medium or iron-restricted Chelex-THY medium at equal inoculation at 37 ^∘^C with 5% CO_2_, OD_600_ was monitored every 2 h for 12 h. All data was conducted using GraphPad Prism 5.0.

### Purification of the SPD_1609 Protein, Preparation of the SPD_1609 Polyclonal Antibody and Western Blotting Analysis

The *spd_1609* gene encoding the SPD_1609 protein without a signal peptide in the *S. pneumoniae* D39 strain was amplified by PCR and ligated into the prokaryotic expression vector pGEX-4T-1 to generate the recombinant plasmid pGEX-4T-1609. The fusion protein GST-1609 was induced by IPTG expression in Escherichia coli BL21 (DE3) and purified by GST affinity chromatography. After the GST-tag was cleaved by thrombin, the SPD_1609 protein was obtained using GST affinity chromatography. Balb/c mice were immunized with the purified SPD_1609 protein without a GST tag to generate SPD_1609 polyclonal antibodies according to the previous study (Zhang et al., 2015). For Western blotting analysis, bacteria were collected when the OD_600_ reached 0.5 and lysed in SDS lysis buffer with sonication. After separation on a 12% SDS-polyacrylamide gel (SDS-PAGE), protein samples were transferred onto polyvinylidene difluoride (PVDF) membranes (Bio-Rad, United States). The membranes were then blocked with 5% (w/v) skim milk and probed with the mouse primary antibody SPD_1609. Horseradish peroxidase (HRP)-conjugated anti-mouse IgG (Promega, United States) was used as the secondary antibody, and the results were visualized using Clarity Western ECL Substrate (Bio-Rad, United States). SDS-PAGE of total proteins was used as the loading control for the Western blotting experiments.

### Inductively Coupled Plasma-Mass Spectrometry (ICP-MS) Analysis

*Streptococcus pneumoniae* D39 wild type, *1609*- mutant and *1609* complement strains were grown in normal THY medium or iron-restricted Chelex-THY medium, respectively. The logarithmic phase cultures were centrifuged and washed three times with prechilled phosphate-buffered saline (PBS) that had been treated with Chelex-100. The cell pellets were dried in a Scanvac Freeze Dryer (Labgene Scientific, Switzerland), and the dry weights were measured. The dried cells were digested in neat trace metal-grade nitric acid for 20 min at 95^∘^C. The acid solution was diluted with ultrapure water and centrifuged at 13,200 g for 30 min. The supernatants were collected and submitted for ICP-MS analysis. The results were expressed as ng of Fe per mg dry weight of cells (Jacobsen et al., 2011; Waterman et al., 2012). Three independent biological experiments were repeated.

### Cell Culture, Adherence and Invasion Assays

A549 human alveolar epithelial cell lines (ATCC: CCL-185) were maintained in DMEM medium (Life Tech Technologies, United States) supplemented with 10% FBS (Gibco, United States) and incubated at 37 ^∘^C in 5% CO_2_. Cells were transferred to 24-well plates and cultivated to confluent cell layers (∼2 × 10^5^ cells/well) for adherence and invasion assays. Bacterial adherence and invasion assays were performed essentially as previously described with minor modifications (Quin et al., 2007; Johnson et al., 2015). Briefly, *S. pneumoniae* wild type, *1609*- mutant and *1609* complement strains were grown to an optical density (OD_600_) of approximately 0.3 and then diluted to 2 × 10^7^ CFU/mL with phenol red-free 1640 medium containing 1% FBS before adding to the monolayer at a multiplicity of infection (MOI) = 100 bacteria/cell and incubated for 1 h (adherence assays) or 2 h (invasion assays).

For adherence assays, the unbound bacteria were removed by washing with 1 × PBS three times, and the number of adhering bacteria was counted by lysing the A549 cells with 0.025% Triton X-100 and plating the lysate. For invasion assays, the monolayers were initially treated as the process for the adherence assay, but after removing the medium and washing with 1 × PBS three times, 1 mL of phenol red-free 1640 medium with 1% FBS containing 100 μg/mL gentamicin was added to the monolayers to kill bacteria that attached to the surfaces of the A549 cells. The plates were incubated for 1 h at 37^∘^C in 5% CO_2_. After this step, the monolayers were washed three times with 1 × PBS, and the number of invading bacteria was counted by lysing the A549 cells with 0.025% Triton X-100 and plating the lysate.

All adherence and invasion experiments were performed in triplicate and repeated three times, and the adherence and invasion abilities were expressed by determining the number of adherent or invasive bacteria per number of host cells.

### Virulence of the *spd-1609*- Mutant in the Mouse Model of Bacteremia Infection

Six-week-old female Balb/c mice were used for animal infection experiments. For the bacteremia infection model, mice were challenged with 5 × 10^6^ CFU of bacteria by intravenous (i.v.) injection through the tail vein and monitored for mortality over the next 14 days. Blood samples were collected via tail venous puncture at 24 h postinfection and properly diluted and plated in replicates on Columbia blood agar plates.

### Real-Time Quantitative PCR (RT-qPCR)

When wild-type *S. pneumoniae*, *1609*- mutant and *1609* complement strains were grown to logarithmic growth phase (OD_600_ = 0.3), total RNA from each strain was extracted with TRIzol reagent (Invitrogen, United States) following the manufacturer’s protocol. The purity and concentration of the isolated RNA were determined using a NanoDrop 2000 UV-VIS Spectrophotometer (Thermo Scientific, United States). cDNA was generated from 1 μg RNA without DNA contamination using the TransScript One-Step gDNA Removal and cDNA Synthesis SuperMix Kit (TransGen Biotech, China) according to the kit’s specifications. RT-qPCR was carried out using EvaGreen Dye (Bio-Rad, United States) in a Miniopticon Real-Time PCR System (Bio-Rad, United States). The cycle threshold (Ct) value was recorded, and the relative quantification of specific gene expression was calculated using the 2^–ΔΔ*Ct*^ method (Livak and Schmittgen, 2001), with *gyrB* as an internal control. The results are shown as the *1609*- mutant or *1609* complement against the wild-type (WT) strain. The primer sequences are shown in Supplementary Table S1. All data were evaluated with three independent biological experiments.

### Histological Analysis

To characterize histopathology following bacteremia infection, lung biopsy samples were collected from mice infected with *S. pneumoniae* WT, *1609-* mutant and *1609* complement strains. Mice were sacrificed at 48 h postinfection, and samples were collected and fixed in 4% paraformaldehyde. Fixed lung samples were embedded in paraffin according to procedures used for routine histology. Stained samples were examined using an Olympus microscope (Olympus, Japan).

### Statistical Analysis

Data from the ICP-MS, RT-qPCR, adhesion and invasion assays were analyzed using two-tailed unpaired Student’s *t*-test and expressed as the mean ± SEM. Data on the survival of mice for the virulence experiment were analyzed using the log-rank (Mantel-Cox) test, and the numbers of CFUs in the different experimental groups were compared using the Mann-Whitney test. Statistical analysis was carried out using GraphPad Prism 5.0, and significant differences were considered when *p* < 0.05.

## Results

### Multiple Amino Acid Sequence Alignment Analysis

**FIGURE 1:**
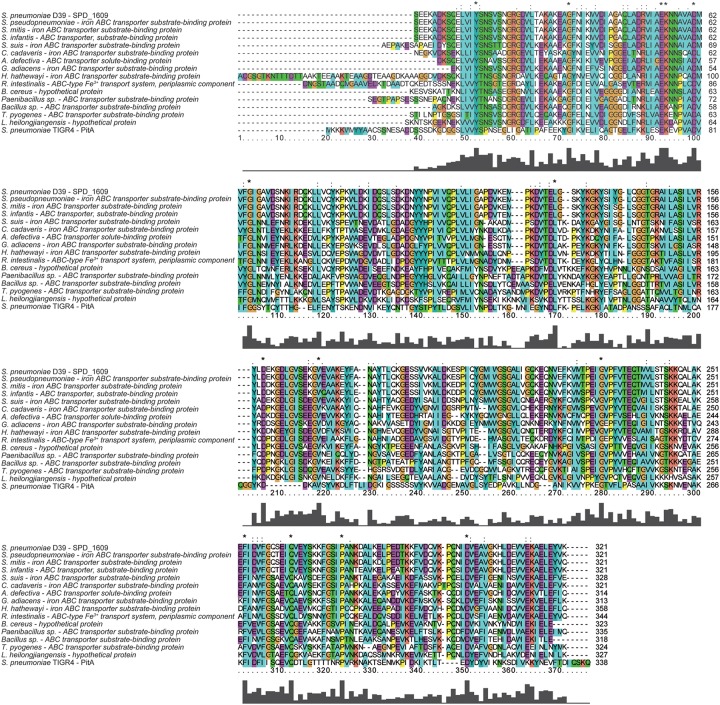
Multiple amino acid sequence alignment of SPD_1609 with proteins belonging to (iron) ABC transporter substrate-binding proteins. Homologous sequences with 40–100% similarities. The residues labeled with ^*^ represent the invariant residues. The height in the bar graph represents the conservation of amino acids.

To discover whether the SPD_1609 protein is conserved in gram-positive bacteria, an alignment was performed using (iron) ABC transporter substrate-binding proteins from *S. pseudopneumoniae*, *S. mitis*, *S. infantis*, *S. suis*, *C. cadaveris*, *A. defective*, *G. adiacens*, *H. hathewayi*, *R. intestinalis*, *B. cereus*, *Paenibacillus* sp., *Bacillus* sp., *T. pyogenes*, and *L. heilongjiangensis* with PSI-BLAST search and Clustal X2.1 soft. The alignment indicated that the sequences are homologous with more than 56% similarities (Table 1). As shown in Figure 1, 14 amino acids in the SPD_1609 protein are invariant residues in all included homologous proteins, with two glutamic acid (E), a tyrosine (Y) and an aspartic acid (D) being the iron-coordination residues in all well-characterized iron binding proteins (Sun et al., 2009; Cheng et al., 2013). In addition, SPD_1609 has no significant similarity with PiaA or PiuA but shares 23% identity and 40% positive with PitA when aligned with the BLAST 2 sequence program (Table 1 and Figure 1).

**TABLE 1:** List of bacterial species and NCBI accession numbers used to perform the alignment of amino acid identities and similarities with the SPD_1609 protein.

**Bacteria**	**Accession numbers**	**Identities with SPD_1609**	**Positives with SPD_1609**
*S. pseudopneumoniae*	WP_023941553.1	99%	100%
*S. mitis*	WP_000738379.1	95%	96%
*S. infantis*	EFO54387.1	92%	95%
*S. suis*	WP_014636980.1	56%	75%
*C. cadaveris*	WP_027639640.1	51%	73%
*A. defective*	WP_023392505.1	51%	72%
*G. adiacens*	WP_005605248.1	53%	71%
*H. hathewayi*	WP_034534116.1	46%	70%
*R. intestinalis*	CBL12437.1	41%	66%
*B. cereus*	WP_000802851.1	43%	67%
*Paenibacillus* sp.	WP_015736942.1	37%	61%
*Bacillus* sp.	WP_028389955.1	38%	62%
*T. pyogenes*	WP_038567276.1	38%	56%
*L. heilongjiangensis*	WP_041501852.1	36%	60%
*S. pneumoniae* TIGR4	AAK74422.1	23%	40%

### Effect of the SPD_1609 Protein on Bacterial Growth

**FIGURE 2:**
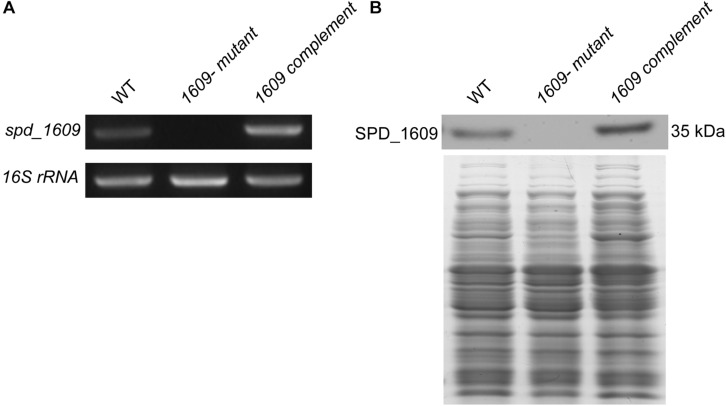
The construction and verification of the *1609-* mutant and *1609* complement strains. **(A)** Verification of the *1609*- mutant and *1609* complement strains using PCR. 16S rRNA was used as a reference. **(B)** Verification of the *1609*- mutant and *1609* complement strains by using Western blotting. SDS-PAGE of total proteins was used as the loading control for the Western blotting experiments.

**FIGURE 3:**
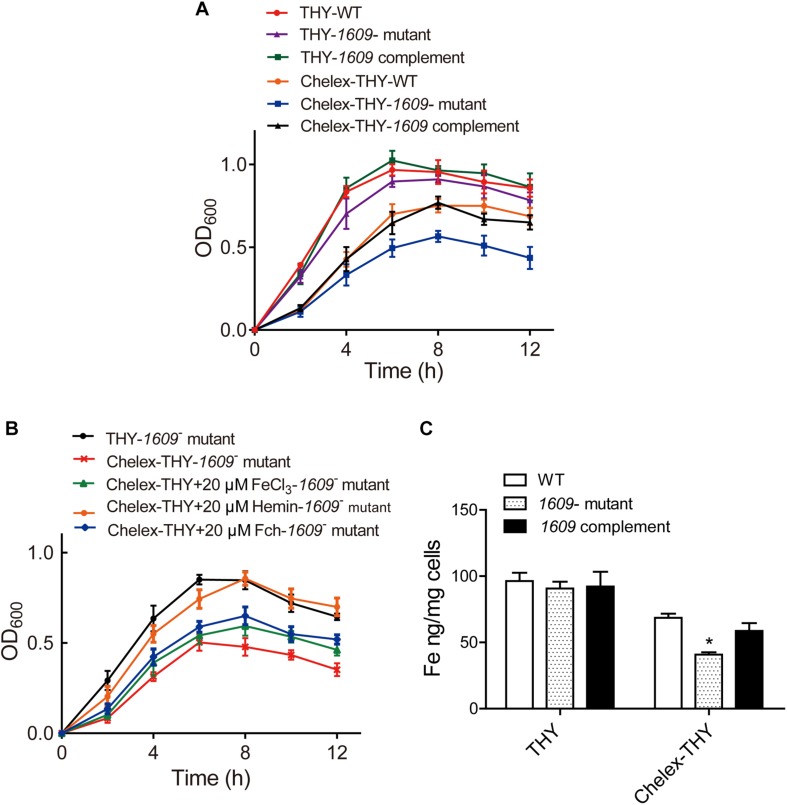
The SPD_1609 protein is required for bacterial growth and iron acquisition under iron-depleted conditions. **(A)** Growth curves of the WT, *1609-* mutant strain and *1609* complement strains under iron-abundant (THY) and iron-depleted conditions (Chelex-THY). **(B)** Growth curves of the *1609-* mutant strain in various media with or without iron. Data are presented as the mean ± SEM from three independent growth curves. **(C)** ICP-MS analysis of Fe content among the WT, *1609-* mutant and *1609* complement strains under the iron-abundant (Normal THY) and iron-depleted conditions (Chelex-THY). ^*^p < 0.05 was used for comparison by Student’s *t*-test to the results vs. WT strain.

To explore the function of the SPD_1609 protein in *S. pneumoniae*, we constructed a *1609*- mutant strain and a *1609* complement strain. The *1609*- mutant and complement strains were confirmed by PCR and Western blotting (Figure 2 and Supplementary Figure S1). First, we detected the growth curves of the WT, *1609-* mutant and *1609* complement strains under iron-abundant (THY) and iron-depleted conditions (Chelex-THY). As shown in Figure 3A, under the iron abundant (THY) condition, the growth curves showed almost no difference among the WT, *1609-* mutant and *1609* complement strains. However, under iron-depleted conditions (Chelex-THY), compared with WT, the *1609-* mutant strain grew slowly and had a lower maximal OD value, while the *1609* complement strain showed similar growth curves to WT. Then, we tested the growth of the *1609*- mutant strain in normal THY media, Chelex-100 treated iron-restricted media (Chelex-THY), and iron-restricted media with the addition of 20 μM FeCl_3_, hemin or ferrichrome (Fch). The growth of the *1609*- mutant strain in iron-restricted media was reduced compared with that in normal THY media, and the addition of hemin could restore growth to a similar normal level; however, the addition of FeCl_3_ or Fch did not restore growth (Figure 3B). These results indicated that lipoprotein SPD_1609 may be involved in FeCl_3_ or ferrichrome uptake but not hemin uptake.

### Effect of the SPD_1609 Protein on Iron Uptake

Although our previous study reported that the *piaA/piuA/1609* triple mutant resulted in impaired iron acquisition compared to the *piaA/piuA* double mutant (Yang et al., 2016), the effect of the *1609*- single mutant on iron uptake is unknown. Therefore, the intracellular levels of iron among the WT, *1609*- mutant and *1609* complement strains were detected using ICP-MS. In normal THY media, the iron contents were not significantly different among the WT, *1609*- mutant and *1609* complement strains (Figure 3C). However, in the iron-depleted media, the iron content in the *1609*- mutant strain was obviously lower than that in the WT and *1609* complement strains (Figure 3C).

### Effect of the SPD_1609 Protein on Adherence and Invasion of the Bacterium to Host Cells

**FIGURE 4:**
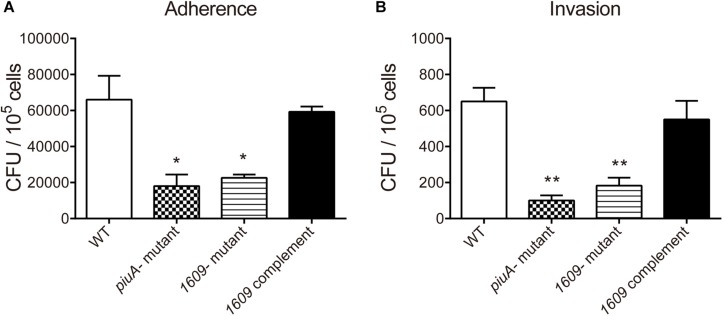
The SPD_1609 protein promotes *S. pneumoniae* to adhere to and invade A549 lung epithelial *in vitro*. **(A)** Decreased A549 cell adherence by the *1609-* mutant strain compared with the WT parent strain was restored by complementation with a 1609-expressing plasmid (pIB169-*1609*). **(B)** Decreased A549 cell invasion by the *1609-* mutant strain compared with the WT parent strain was restored by complementation with the pIB169-*1609* plasmid. The *piuA-* mutant as a positive control. Adherence and invasion assays were performed in triplicate and repeated three times; ^*^p < 0.05, ^∗∗^*p* < 0.01 vs. WT parent strain by Student’s *t*-test analysis.

In addition to pneumolysin and the phagocytosis-inhibiting polysaccharide capsule, the virulence of *S. pneumoniae* is enhanced by the capacity of bacteria to adhere to and invade host cells and then diffusion into host tissue (Andrews et al., 2003; Zakrzewicz et al., 2016). Many surface proteins of *S. pneumoniae* have been shown to be involved in adherence and invasion processes (Balachandran et al., 2002; Uchiyama et al., 2009; Keller et al., 2013). Considering that SPD_1609 is a surface lipoprotein, we investigated whether SPD_1609 could affect pneumococcal adherence and invasion to human alveolar epithelial cells (A549) in vitro, and *piuA-* mutant as a positive control. As shown in Figure 4, the *1609-* mutant strain exhibited an almost 60% decrease in A549 adherence and a 70% decrease in A549 invasion compared with the WT parent strain, and the defect was rescued upon complementation of the mutant with *spd-1609* on a plasmid vector: *1609* complement strain. This result indicates that SPD_1609 is important for *S. pneumoniae* colonization of human lung carcinoma cells.

### Gene Expression of *S. pneumoniae* Regulated by SPD_1609

**FIGURE 5:**
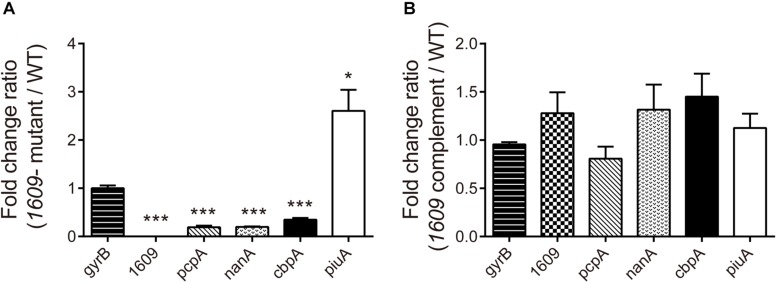
Influence of gene expression involved in colonization of the *1609-* mutant strain. RT-qPCR expression analysis of the *pcpA*, *cbpA*, *nanA*, *piuA*, and *spd_1609* genes in the WT, *1609*- mutant and *1609* complement strains. The relative gene expression was calculated with *gyrB* as the reference gene. All results represent the relative expression level of the *1609*- mutant strain vs. the WT strain **(A)** or the *1609* complement strain vs. the WT strain **(B)**, shown as the mean value (±SEM) from three independent biological experiments. ^*^p < 0.05, ^∗∗∗^*p* < 0.001 compared to *gyrB* as determined by Student’s *t*-test analysis.

To further explore the reason that the *1609*- mutation reduced the adherence and invasion of the bacterium to host A549 cells, the gene expression of a range of surface proteins including adhesins choline binding protein A (*pcpA* and *cbpA*), neuraminidase A (*nanA*) and *piuA* involved in colonization (Sanchez-Beato et al., 1998; Kadioglu et al., 2008) was detected using RT-qPCR. The RT-qPCR results indicated that of the *pcpA* and *cbpA* and *nanA* genes were downregulated in the *1609*- mutant strain compared with the WT parent strain (Figure 5A), consistent with the observation that the *spd-1609* deletion reduced bacterial adherence and invasion to host A549 cells. However, *piuA* was induced in the *1609*- mutant strain compared to WT; one possible reason for the high expression of *piuA* in the *1609*- mutant strain could be to compensate for iron uptake. In contrast, these genes showed no significant change between the WT and *1609* complement strains (Figure 5B).

### Importance of the SPD_1609 Protein for *S. pneumoniae* Virulence *in vivo*

**FIGURE 6:**
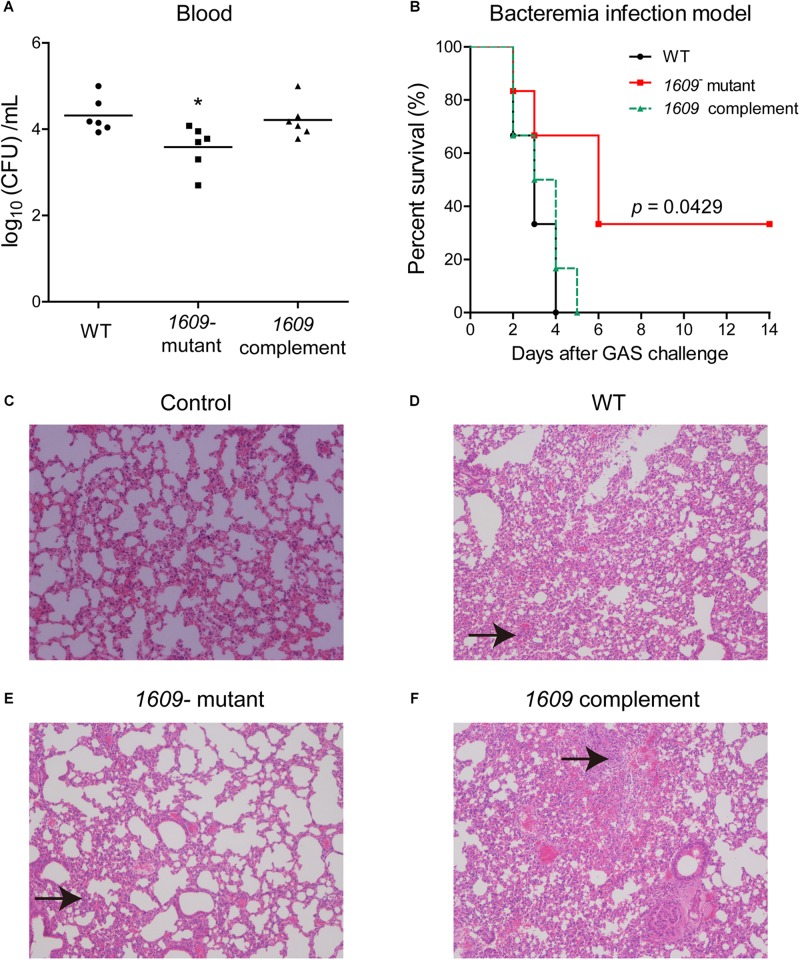
The SPD_1609 protein is required for virulence during bacteremia infection. **(A)** Bacterial burdens of the WT, *1609*- mutant and *1609* complement strains in the bloodstream of mice 24 h post-challenge. Horizontal lines represent median values. ^*^p < 0.05 for comparison by Mann-Whitney test to the results for mice infected with the parental WT; *n* = 6 animals per group. **(B)** Survival curves of mice infected with 5 × 10^6^ CFU of WT, *1609*- mutant or *1609* complement strains by intravenous (i.v.) injection through the tail vein; *n* = 6 animals per group. Survival was analyzed using the log-rank (Mantel-Cox) test between the WT and *1609*- mutant strains. **(C–F)** Representative microphotographs of HE-stained lung sections of mice uninfected with bacteria (control), and mice infected with WT, *1609*- mutant or *1609* complement strains (10 × original magnification). Black arrows indicate the inflammatory cells.

We also dissected the importance of the SPD_1609 protein *in vivo*, and a mouse model of bacteremia infection was employed. For bacteremia infection, a group of six mice was inoculated i.v. with 5 × 10^6^ CFU of the WT, the *1609*- mutant or the *1609* complement strain through the tail vein and monitored for mortality over a 14-day period. Mice infected with the *1609*- mutant had significantly lower bacterial CFU in the blood at 24 h following infection than the mice infected with the WT or the *1609* complement strains (Figure 6A). Moreover, the mice infected with the *1609*- mutant displayed significantly higher survival rates compared with the mice infected with the WT strain (Figure 6B). When the *1609*- mutant strain was complemented with the *spd_1609* gene, no significant difference was observed in survival (Figure 6B) or bacterial blood titers (Figure 6A) compared to the results for infection with the WT strain.

To further observe the effect of the SPD_1609 protein on bacteremia infection, histology analysis was performed. The lung biopsy samples were collected from the mice uninfected with bacteria (control), and the mice infected with wild-type *S. pneumoniae*, *1609*- mutant and *1609* complement strains after 48 h of infection. Lung histological examination revealed that mice infected with WT, *1609*- mutant or *1609* complement strains displayed alveolar disruption, with more inflammatory cell infiltration than control (Figures 6C–F). However, less alveolar damage and inflammatory cell infiltration was observed in mice infected with the *1609*- mutant when compared with mice infected with the WT or *1609* complement strains (Figures 6D–F).

These results highlight the importance of the SPD_1609 protein for virulence in a mouse model of bacteremia infection, indicating that the SPD_1609 lipoprotein is important for bacteremia infection during *in vivo* growth.

## Discussion

Iron acquisition is key for the growth and infection of *S. pneumoniae*. Three well-known ABC transporters found in *S. pneumoniae*, PiaABC, PiuABC and PitABC, are involved in iron uptake. We recently reported that lipoprotein SPD_1609 in operon 804 is involved in iron uptake. In the present study, we demonstrated that lipoprotein SPD_1609 is conserved in a variety of gram-positive bacteria (Figure 1). ICP-MS data indicated that the *1609*- mutant strain impaired iron uptake only in iron-depleted media, and growth curve assays indicated that lipoprotein SPD_1609 may be involved in FeCl_3_ or ferrichrome uptake, but not hemin uptake (Figure 3).

Further experiments revealed that the *1609*- mutant strain has a significantly lower ability to adhere to and invade host cells than the WT strain (Figure 4). RT-qPCR assays indicated that deletion of the *spd_1609* gene resulted in decreased gene expression involved in colonization. Moreover, at 24 h after bacteremia infection, the bacteria in the blood of mice infected with the *1609*- mutant strain were markedly reduced compared to the infection with the WT strain (Figure 6A). This result corresponds to a significant increase in the survival of mice infected with the *1609*- mutant strain compared with the survival of mice infected with the WT or *1609* complement strains (Figure 6B). Taken together, these data revealed that lipoprotein SPD_1609 contributes to bacterial virulence during bacteremia infection.

Previous reports have suggested that the *piaA*- mutation attenuated bacterial virulence in both systemic and pulmonary infection models, and both *piuB*- and *pitA*- mutants exhibited reduced virulence only in the systemic infection model (Brown et al., 2001a, 2002). In this study, the *1609*- mutation showed decreased bacterial virulence in the bacteremia infection model, which once again confirmed that the iron acquisition systems are associated with pneumococcal virulence.

Several bacterial surface proteins and lipoproteins of iron acquisition systems have elicited protective immunity in mice and thus are used as candidate vaccine antigens (Jomaa et al., 2006; Schmaler et al., 2009; Kim et al., 2010; Zhang et al., 2017). Jomaa et al. (2006) reported that immunization with PiaA and PiuA elicits specific antibody responses that prevent respiratory infection with *S. pneumoniae*. In *S. aureus*, cell wall-anchored surface proteins IsdA and IsdB of the iron-regulated surface determinants (Isd) system antibodies protect mice against abscess formation and lethal challenge (Kim et al., 2010). The staphylococcal surface lipoprotein FhuD2 (ferric-hydroxamate uptake D2), a component of an Fe ABC transporter, was considered to be a potential vaccine candidate as a protective antigen in a murine staphylococcal infection model (Mariotti et al., 2013; Shahmirzadi et al., 2016). In *S. pyogenes*, vaccination of surface protein Shp of the heme acquisition system protects mice against skin formation and lethal challenge (Zhang et al., 2017). In this connection, lipoprotein SPD_1609 may be a vaccine candidate because of its participation in iron acquisition and its contribution to virulence during bacteremia infection. Accordingly, our future work would investigate whether SPD_1609 can be a useful vaccine antigen for preventing *S. pneumoniae* infection and whether it can be more effective in combination with PiaA and PiuA or other novel *S. pneumoniae* vaccine candidates.

## Data Availability

The raw data supporting the conclusions of this manuscript will be made available by the authors, without undue reservation, to any qualified researcher.

## Author Contributions

X-YY designed the research, performed the experimental work, and wrote the manuscript. NL performed the experimental work. J-YX and XS analyzed the data. Q-YH provided the initial idea and designed the research.

## Conflict of Interest Statement

The authors declare that the research was conducted in the absence of any commercial or financial relationships that could be construed as a potential conflict of interest.

## Supplementary Material

FIGURE S1 RT-qPCR expression analysis of the genes *spd_1608*, *spd_1609*, *spd_1610* in WT and *1609*- mutant strain.

TABLE S1 The primer sequences of genes from *Streptococcus pneumoniae* D39 (GenBank: CP000410.2).

## References

[B1] Andrews S. C. Robinson A. K. Rodriguez-Quinones F. (2003). Bacterial iron homeostasis. *FEMS Microbiol. Rev.* 27 215–237. 10.1016/s0168-6445(03)00055-x 12829269

[B2] Balachandran P. Brooks-Walter A. Virolainen-Julkunen A. Hollingshead S. K. Briles D. E. (2002). Role of pneumococcal surface protein C in nasopharyngeal carriage and pneumonia and its ability to elicit protection against carriage of *Streptococcus pneumoniae*. *Infect. Immun.* 70 2526–2534. 10.1128/iai.70.5.2526-2534.2002 11953392 PMC127914

[B3] Biswas I. Jha J. K. Fromm N. (2008). Shuttle expression plasmids for genetic studies in *Streptococcus mutans*. *Microbiology* 154 2275–2282. 10.1099/mic.0.2008/019265-0 18667560 PMC4110107

[B4] Brown J. S. Gilliland S. M. Holden D. W. (2001a). A *Streptococcus pneumoniae* pathogenicity island encoding an ABC transporter involved in iron uptake and virulence. *Mol. Microbiol.* 40 572–585. 10.1046/j.1365-2958.2001.02414.x 11359564

[B5] Brown J. S. Ogunniyi A. D. Woodrow M. C. Holden D. W. Paton J. C. (2001b). Immunization with components of two iron uptake ABC transporters protects mice against systemic *Streptococcus pneumoniae* infection. *Infect. Immun.* 69 6702–6706. 10.1128/iai.69.11.6702-6706.2001 11598041 PMC100046

[B6] Brown J. S. Gilliland S. M. Ruiz-Albert J. Holden D. W. (2002). Characterization of pit, a *Streptococcus pneumoniae* iron uptake ABC transporter. *Infect. Immun.* 70 4389–4398. 10.1128/iai.70.8.4389-4398.2002 12117949 PMC128127

[B7] Cassat J. E. Skaar E. P. (2013). Iron in infection and immunity. *Cell Host Microbe* 13 509–519. 10.1016/j.chom.2013.04.010 23684303 PMC3676888

[B8] Cheng W. Li Q. Jiang Y. L. Zhou C. Z. Chen Y. (2013). Structures of *Streptococcus pneumoniae* PiaA and its complex with ferrichrome reveal insights into the substrate binding and release of high affinity iron transporters. *PLoS One* 8:e71451. 10.1371/journal.pone.0071451 23951167 PMC3741162

[B9] Honsa E. S. Johnson M. D. Rosch J. W. (2013). The roles of transition metals in the physiology and pathogenesis of *Streptococcus pneumoniae*. *Front. Cell. Infect. Microbiol.* 3:92. 10.3389/fcimb.2013.00092 24364001 PMC3849628

[B10] Hutchings M. I. Palmer T. Harrington D. J. Sutcliffe I. C. (2009). Lipoprotein biogenesis in Gram-positive bacteria: knowing when to hold ’em, knowing when to fold ’em. *Trends Microbiol.* 17 13–21. 10.1016/j.tim.2008.10.001 19059780

[B11] Jacobsen F. E. Kazmierczak K. M. Lisher J. P. Winkler M. E. Giedroc D. P. (2011). Interplay between manganese and zinc homeostasis in the human pathogen *Streptococcus pneumoniae*. *Metallomics* 3 38–41. 10.1039/c0mt00050g 21275153 PMC3061551

[B12] Johnson M. D. Kehl-Fie T. E. Klein R. Kelly J. Burnham C. Mann B. (2015). Role of copper efflux in pneumococcal pathogenesis and resistance to macrophage-mediated immune clearance. *Infect. Immun.* 83 1684–1694. 10.1128/IAI.03015-14 25667262 PMC4363445

[B13] Jomaa M. Terry S. Hale C. Jones C. Dougan G. Brown J. (2006). Immunization with the iron uptake ABC transporter proteins PiaA and PiuA prevents respiratory infection with *Streptococcus pneumoniae*. *Vaccine* 24 5133–5139. 10.1016/j.vaccine.2006.04.012 16707196

[B14] Kadioglu A. Weiser J. N. Paton J. C. Andrew P. W. (2008). The role of *Streptococcus pneumoniae* virulence factors in host respiratory colonization and disease. *Nat. Rev. Microbiol.* 6 288–301. 10.1038/nrmicro1871 18340341

[B15] Keller L. E. Jones C. V. Thornton J. A. Sanders M. E. Swiatlo E. Nahm M. H. (2013). PspK of *Streptococcus pneumoniae* increases adherence to epithelial cells and enhances nasopharyngeal colonization. *Infect. Immun.* 81 173–181. 10.1128/IAI.00755-12 23115034 PMC3536125

[B16] Kim H. K. Dedent A. Cheng A. G. Mcadow M. Bagnoli F. Missiakas D. M. (2010). IsdA and IsdB antibodies protect mice against *Staphylococcus aureus* abscess formation and lethal challenge. *Vaccine* 28 6382–6392. 10.1016/j.vaccine.2010.02.097 20226248 PMC3095377

[B17] Kohler S. Voss F. Gomez Mejia A. Brown J. S. Hammerschmidt S. (2016). Pneumococcal lipoproteins involved in bacterial fitness, virulence, and immune evasion. *FEBS Lett.* 590 3820–3839. 10.1002/1873-3468.12352 27508940

[B18] Kovacs-Simon A. Titball R. W. Michell S. L. (2011). Lipoproteins of bacterial pathogens. *Infect. Immun.* 79 548–561. 10.1128/IAI.00682-10 20974828 PMC3028857

[B19] Livak K. J. Schmittgen T. D. (2001). Analysis of relative gene expression data using real-time quantitative PCR and the 2(-Delta Delta C(T)) Method. *Methods* 25 402–408. 10.1006/meth.2001.1262 11846609

[B20] Mariotti P. Malito E. Biancucci M. Lo Surdo P. Mishra R. P. Nardi-Dei V. (2013). Structural and functional characterization of the *Staphylococcus aureus* virulence factor and vaccine candidate FhuD2. *Biochem. J.* 449 683–693. 10.1042/BJ20121426 23113737

[B21] Nguyen M. T. Gotz F. (2016). Lipoproteins of gram-positive bacteria: key players in the immune response and virulence. *Microbiol. Mol. Biol. Rev.* 80 891–903. 10.1128/MMBR.00028-16 27512100 PMC4981669

[B22] Pishchany G. Sheldon J. R. Dickson C. F. Alam M. T. Read T. D. Gell D. A. (2014). IsdB-dependent hemoglobin binding is required for acquisition of heme by *Staphylococcus aureus*. *J. Infect. Dis.* 209 1764–1772. 10.1093/infdis/jit817 24338348 PMC4038968

[B23] Quin L. R. Onwubiko C. Moore Q. C. Mills M. F. Mcdaniel L. S. Carmicle S. (2007). Factor H binding to PspC of *Streptococcus pneumoniae* increases adherence to human cell lines in vitro and enhances invasion of mouse lungs in vivo. *Infect. Immun.* 75 4082–4087. 10.1128/iai.00474-07 17562771 PMC1952001

[B24] Romero-Espejel M. E. Gonzalez-Lopez M. A. Olivares-Trejo Jde J. (2013). *Streptococcus pneumoniae* requires iron for its viability and expresses two membrane proteins that bind haemoglobin and haem. *Metallomics* 5 384–389. 10.1039/c3mt20244e 23487307

[B25] Sanchez-Beato A. R. Lopez R. Garcia J. L. (1998). Molecular characterization of PcpA: a novel choline-binding protein of Streptococcus pneumoniae. *FEMS Microbiol. Lett.* 164 207–214. 10.1016/s0378-1097(98)00206-7 9675866

[B26] Schaible U. E. Kaufmann S. H. (2004). Iron and microbial infection. *Nat. Rev. Microbiol.* 2 946–953. 10.1038/nrmicro1046 15550940

[B27] Schmaler M. Jann N. J. Ferracin F. Landolt L. Z. Biswas L. Gotz F. (2009). Lipoproteins in *Staphylococcus aureus* mediate inflammation by TLR2 and iron-dependent growth in vivo. *J. Immunol.* 182 7110–7118. 10.4049/jimmunol.0804292 19454708

[B28] Shahmirzadi S. V. Nguyen M. T. Gotz F. (2016). Evaluation of *Staphylococcus aureus* lipoproteins: role in nutritional acquisition and pathogenicity. *Front. Microbiol.* 7:1404. 10.3389/fmicb.2016.01404 27679612 PMC5020093

[B29] Sun X. Baker H. M. Ge R. Sun H. He Q. Y. Baker E. N. (2009). Crystal structure and metal binding properties of the lipoprotein MtsA, responsible for iron transport in *Streptococcus pyogenes*. *Biochemistry* 48 6184–6190. 10.1021/bi900552c 19463017

[B30] Torres V. J. Pishchany G. Humayun M. Schneewind O. Skaar E. P. (2006). *Staphylococcus aureus* IsdB is a hemoglobin receptor required for heme iron utilization. *J. Bacteriol.* 188 8421–8429. 10.1128/jb.01335-06 17041042 PMC1698231

[B31] Turner A. G. Ong C. Y. Walker M. J. Djoko K. Y. Mcewan A. G. (2017). Transition Metal Homeostasis in *Streptococcus pyogenes* and *Streptococcus pneumoniae*. *Adv. Microb. Physiol.* 70 123–191. 10.1016/bs.ampbs.2017.01.002 28528647

[B32] Uchiyama S. Carlin A. F. Khosravi A. Weiman S. Banerjee A. Quach D. (2009). The surface-anchored NanA protein promotes pneumococcal brain endothelial cell invasion. *J. Exp. Med.* 206 1845–1852. 10.1084/jem.20090386 19687228 PMC2737157

[B33] Van Der Poll T. Opal S. M. (2009). Pathogenesis, treatment, and prevention of pneumococcal pneumonia. *Lancet* 374 1543–1556. 10.1016/S0140-6736(09)61114-4 19880020

[B34] Wach A. (1996). PCR-synthesis of marker cassettes with long flanking homology regions for gene disruptions in *S. cerevisiae*. *Yeast* 12 259–265. 10.1002/(sici)1097-0061(19960315)12:3<259::aid-yea901>3.0.co;2-c 8904338

[B35] Waterman S. R. Park Y. D. Raja M. Qiu J. Hammoud D. A. O’halloran T. V. (2012). Role of CTR4 in the virulence of *Cryptococcus neoformans*. *MBio* 3 e00285–12. 10.1128/mBio.00285-12 23033470 PMC3518914

[B36] Yang X. Y. He K. Du G. Wu X. Yu G. Pan Y. (2016). Integrated translatomics with proteomics to identify novel iron-transporting proteins in *Streptococcus pneumoniae*. *Front. Microbiol.* 7:78. 10.3389/fmicb.2016.00078 26870030 PMC4738293

[B37] Zakrzewicz D. Bergmann S. Didiasova M. Giaimo B. D. Borggrefe T. Mieth M. (2016). Host-derived extracellular RNA promotes adhesion of *Streptococcus pneumoniae* to endothelial and epithelial cells. *Sci. Rep.* 6:37758. 10.1038/srep37758 27892961 PMC5125276

[B38] Zhang B. Z. Hua Y. H. Yu B. Lau C. C. Y. Cai J. P. Zheng S. Y. (2015). Recombinant ESAT-6-like proteins provoke protective immune responses against invasive *Staphylococcus aureus* disease in a murine model. *Infect. Immun.* 83 339–345. 10.1128/IAI.02498-14 25368117 PMC4288882

[B39] Zhang X. Song Y. Li Y. Cai M. Meng Y. Zhu H. (2017). Immunization with streptococcal heme binding protein (Shp) protects mice against group A streptococcus infection. *Adv. Exp. Med. Biol.* 973 115–124. 10.1007/5584_2016_198 28190144

