# Supplementary material for: Lipoprotein SPD_1609 of Streptococcus pneumoniae Promotes Adherence and Invasion to Epithelial Cells Contributing to Bacterial Virulence
Source: Front Microbiol. 2019 Jul 30;10:1769. doi: 10.3389/fmicb.2019.01769 (PMC6682666; doi:10.3389/fmicb.2019.01769)
Supplement: TABLE S1 — The primer sequences of genes from Streptococcus pneumoniae D39 (GenBank: CP000410.2). [file Table_1.DOCX]

**Table S1.** The primer sequences of genes from *Streptococcus pneumoniae* D39 (GenBank: CP000410.2).

| Primer | Sequence (5’–3’) | |
| --- | --- | --- |
| *spd-1609*–P1 | | CTTCGTGGACTGTTTCATCG |
| *spd-1609*–P2 | | ATGTAAACATAGTGACACCGA |
| *spd-1609*–P3 | | ATCAAACAAATTTTGGGCCCGG ATTCTTTACATTTCTTGGGC |
| *spd-1609*–P4 | | TCGTTAAGGGATCAACTTTGGGAAGTAAACTCTTCTTCCTCCTC |
| *tet-F* | | CCGGGCCCAAAATTTGTTTGAT |
| *tet-R* | | TCCCAAAGTTGATCCCTTAACGA |
| *pIB169-1609-F* | | ACGGGGCCGGAGACCGCGGTATGAAAAAAATGAAAGTTTGGTC |
| *pIB169-1609-R* | | TCGAGGGATCCCCGGGTACCTTAGTGATGGTGATGGTGATGTTTTACGTATTCTAATTCAGC |
| *gyrB-F* | | TCGTGTGGCTGCCAAGCGTG |
| *gyrB-*R | | GGCTGATCCACCAGCTGAGTC |
| *spd_1609*-F | | TTCATCAAGGTAACGAACTAAGA |
| *spd_1609*-R | | AAGAAATGCCTAAAGATTGGACT |
| *pcpA*-F | | TCTTTTGTCTCCTTAGGCGTTTT |
| *pcpA*-R | | CCTCAGTTGATGGTGTTTTGTTT |
| *nanA*-F | | GAAATCGCAGAGTATAAGG |
| *nanA*-R | | GTAAACAGACCAAGGAAGA |
| *cbpA*-F | | TTGCCAGTCTTGTTATGGGAA |
| *cbpA*-R | | AGGCGACATTTTGGGTATGTT |
| *piuA-F* | | TAGTCAGACAGAGACCAGT |
| *piuA-R* | | CTTTCATAGAACCAACATT |

The sequences underlining of *spd-1609*–P3 and P4 are the reverse complement of tet-F and R. The locus_tags of *gyrB*, *nanA*, *piuA*, *pcpA* and *cbpA* genes are SPD_0709, SPD_1504, SPD_1652, SPD_1965, SPD_2017, respectively.
